# Analysis of risk indicators for prevalence of peri-implant diseases in Turkish population

**DOI:** 10.1186/s40729-020-00215-9

**Published:** 2020-05-20

**Authors:** Sadiye Gunpinar, Bilge Meraci, Mert Karas

**Affiliations:** Department of Periodontology, Faculty of Dentistry, Bolu Abant Izzet Baysal University, Bolu, Turkey

**Keywords:** Peri-implant diseases, Peri-implant mucositis, Peri-implantitis, Keratinized mucosa, Prevalence, Risk indicators

## Abstract

**Background:**

The aim of this cross-sectional study was (1) to determine the prevalence of peri-implant mucositis and peri-implantitis and (2) to reveal the risk indicators associated with peri-implant diseases. The second point was to investigate the role of keratinized mucosa on peri-implant health.

**Materials and methods:**

Three hundred and eighty-two subjects who were treated with 1415 dental implants between 2011–2017 were clinically evaluated. Patients’ medical and dental history, as well as implant details, were recorded. Peri-implant examination included probing pocket depth (PPD), bleeding on probing (BoP), plaque index (PI), gingival index (GI), and keratinized tissue width. Furthermore, the patient (sex, age, and smoking) and implant/prosthesis-related factors (surface characteristic, time in function, design of prosthesis etc.) were evaluated. Implants were classified into three groups: healthy, peri-implant mucositis, and peri-implantitis. Uni- and multi-variate regression analyses were utilized for statistics.

**Results:**

41.1% (*n* = 157) and 36.9% (*n* = 84) of patients had mucositis and peri-implantitis, respectively. 53.6% (*n* = 758) of implants (95%CI 80.2–90.4) had mucositis, and 21.7% (*n* = 307) had peri-implantitis. Patients with a maintenance < 2/year (OR = 2.576), having periodontitis (OR = 3.342) and higher PI (OR = 3.046) had significant associations with the development of peri-implant mucositis. Significant ORs were determined for peri-implantitis with patients having maintenance < 2/year (OR = 2.048), having number of implants ≥ 4 (OR = 2.103), diagnosed with periodontitis (OR = 3.295), and higher PI (OR = 7.055). Keratinized tissue width < 2 mm (ORs = 5389/8.013), PPD (ORs = 1.570/8.338), PI (ORs = 6.726/5.205), and BoP (ORs = 3.645/4.353) independent variables were significantly associated with both peri-implant mucositis and peri-implantitis at implant level, respectively.

**Conclusions:**

Within the limits of this study, the prevalence of mucositis and peri-implantitis was shown to be high in Turkish population. Furthermore, increased risk for peri-implantitis was identified in patients having maintenance < 2/year, presence of periodontitis, poor plaque control, and having number of implants ≥ 4. Less keratinized tissue (< 2 mm), PPD, and BoP were also risk indicators for peri-implantitis development.

## Introductıon

Peri-implant mucositis is identified as an inflammatory state which only affects soft tissue around implants. On the other hand, peri-implantitis affects both soft and hard tissue and is characterized with progressive loss of alveolar bone [1]. There is growing interest for researchers to investigate the peri-implant diseases including both peri-implant mucositis and peri-implantitis because of increasing high prevalence [2–4].

Peri-implant diseases are initiated by microbial dental biofilm similarly to periodontal diseases including gingivitis and periodontitis [1]. Current literature supports that succesful treatment of periodontal diseases can be achieved more handily; however, once the peri-implant supporting tissues are lost, then regeneration of soft and hard tissues could not be possible [5, 6]. Therefore, prevention of peri-implant diseases are more essential and important than treatment, to increase the success rate of the implant for long-term.

Knowledge of the factors that lead to peri-implant disease is crucial for maintaining the dental implants to function properly. Several patient- and implant-related risk indicators including poor oral hygiene, smoking, history of periodontal disease, and compliance of maintenance have been reported [2–4, 7]. On the other hand, the necessity of keratinized tissue around implant is controversial. Some researchers reported that insufficient or an absence of keratinized mucosa (KM) is related to increased plaque accumulation and inflammatory parameters around implants [2, 8–10], others did not find a necessity for KM width to maintain peri-implant health [11–13]. In a systematic review, the presence of at least 1 to 2-mm width of KM might be crucial for maintenance of peri-implant stability [14]. Besides, Wennstrom and Derks [15] reported that the need for keratinized mucosa was limited to maintain peri-implant soft and hard tissue stability. Furthermore, a threshold value for the presence of KM width, whether 1 mm or 2 mm, is not available in the literature. Recently, Lim et al. [13] investigated the influence of KM on peri-implant health and concluded that in compliant subjects, the width of KM may not be necessary to maintain peri-implant health. In addition, they could not identify a thereshold value for the need of KM width.

In the shed of these findings, the purpose of this cross-sectional study is to determine the prevalence of peri-implant diseases and to clarify the probable systemic and local risk indicators related to peri-implant health in subjects treated in a university periodontology clinic.

## Materıals and methods

### Study design

The present cross-sectional study was conducted in Periodontology Department, in Bolu Abant Izzet Baysal University. Patients received dental implant treatment and implant-supported prosthetic rehabilitation between 2011–2016 by one trained and experienced periodontology specialist (SG) and by three research assistants under the supervision of the same experienced periodontology specialist (SG). The patients were recalled for re-evaluations which included clinical examinations since June 2017.

The methodology of the study was approved by the ethics committee in human research from the School of Medicine, Bolu Abant Izzet Baysal University (decision number: 2017/175). Before the study, each patient was verbally informed about the procedures, and signed informed consent was obtained.

### Study population

#### Inclusion and exclusion criteria

Patients (> 18 years old) rehabilitated with one or more dental implants, with at least 12 months of follow-up after loading, had panoramic radiograph immediately after surgery, and received regular or irregular maintenance therapy at the same periodontology department where the implants had been surgically inserted were included in this study. Individuals previously diagnosed with aggressive periodontitis [16]; had inadequate radiograph; had received administration of bisphosphonates and immunosuppressive medications, or antibiotic therapies in the previous 6 months were excluded. Pregnant or lactating women and subjects who had other missing data were also excluded.

### Outcome measures

#### Clinical parameters

For each subject, age, gender, periodontal diagnosis (healthy, gingivitis, periodontitis) (17), presence of systemic diseases (cardiovascular disease, hypertension, or diabetes), drug intake, number of dental implants, and frequency of maintenance (at least two maintenance appointments per year/less than two per year or none) were recorded. Smoking habit was also recorded and categorized as non-smoker (subjects who never smoke or quit smoking for at least 5 years) and current smoker (< 10 cigarettes/day or ≥ 10 cigarettes/day).

For each implant, the following data were collected: location (anterior/posterior, maxilla/mandible), time in function (months), implant brand, type of prosthesis (fixed-multiple/single or removable) and previous bone augmentation procedure (yes/no).

The following peri-implant clinical parameters were recorded: probing pocket depth (PPD) in six sites per implant, plaque index (PI) [18] and gingival index (GI) [19] in four sites per implant (vestibul-lingual/palatinal-mesial-distal), bleeding on probing (BoP) measured as presence/absence at six sites per implant, and width of keratinized mucosa (KM). KM was measured in millimeter at three points (1-mm mesial-midpoint and 1-mm distal of buccal site) between gingival margin and mucogingival line. Mucogingival junction was determined by mobility test, and the mean of those three measurements was recorded as KM (0–2 mm/≥ 2 mm).

Patients with periodontitis were defined as the existence of periodontal pocket depths ≥ 4 mm with BoP and clinical attachment loss > 2 mm in at least two non-adjacent interproximal area [17]. All clinical assessments were performed by a single and previously calibrated investigator (M.K.), using a manual periodontal probe (PCP-UNC 15: Hu-Friedy, Chicago, IL, USA). Intraexaminer calibration were performed in ten individuals not participating in the study presenting peri-implant mucositis and peri-implantitis. PPD values were recorded on six surfaces, and GI scores were recorded on four surfaces of each implant, in two separate sessions, 48 h apart. The intraclass correlation coefficients ranged between 0.811 and 0.923 for PPD, and the Cohen’s kappa (κ) value of GI was 0.824.

### Case definition

Peri-implant health was defined as absence of bleeding and/or suppuration on probing and no detectable radiographic marginal bone loss after initial bone remodeling. Peri-implant mucositis was diagnosed as tissue edema with bleeding on probing and absence of bone loss after initial crestal bone remodeling. On the other hand, diagnosis of peri-implantitis was based on the presence of bleeding and/or suppuration on probing, probing depth > 5 mm, and marginal bone loss > 2 mm reference to the most coronal part of the implant [1].

The implant with the most severe clinical diagnosis was chosen to describe the patient diagnosis. Accordingly, an individual was diagnosed as having peri-implantitis if at least one implant had the diagnosis of peri-implantitis, and an individual was diagnosed as having peri-implant mucositis if one or more implant had the diagnosis of peri-implant mucositis.

### Statistical analysis

Descriptive statistics are presented as frequencies and percentages. The prevalence of peri-implant health, peri-implant mucositis, and peri-implantitis were reported at implant and patient levels. Risk indicators for peri-implant mucositis and peri-implantitis were analyzed. For all models, the odds ratio (OR) and the 95% confidence intervals (CI) were reported.

Univariate logistic regression analysis was conducted for each independent variable individually to determine factors associated with peri-implant mucositis and peri-implantitis. Factors with a combination of *p* values < 0.05 were chosen for the multivariate logistic regression. To avoid multicollinearity, Spearman’s correlation analysis was used. Accordingly, implant-related independent variables GI and BoP (*p* = 0.000, *r* = 0.859) and dental arch and implant position (*p* = 0.000, *r* = 0.908) showed strong correlations. Therefore, after univariate regression, BoP and implant position independent variables were included in the final multivariate regression model. The statistical software SPSS (IBM, Version 22.0, Armonk, NY) was used for all analyses, and statistical differences with *p* values < 0.05 were considered significant. All the statistical results obtained from SPSS were confirmed with R statistics, and the results from SPSS were utilized in the tables.

## Results

### Demografic and clinical evaluation

A group of 424 patients were evaluated for inclusion, out of which, 42 patients were excluded. Among these, 36 had implants with < 1 year of follow-up from prosthetic loading, and six were pregnant. The patient- and implant-related demographics and clinical parameters are shown in Tables 1 and 2. Three hundred eighty two patients (175 male and 207 female) with 1415 dental implants participated and clinically evaluated in this study.

**Table 1:** Patient-related categorical data analyzed in this study

Patient-related data	*N* = 382	
	*n*	%
Gender		
Female/Male	207/175	54.2/45.8
Smoking		
Non-smoker	252	66.0
Smoker (≥ 10)	130	34.0
Systemic disease		
Yes/No	113/269	29.6/70.4
Diabetes		
Yes/No	45/337	11.8/88.2
Periodontal diagnosis		
Healthy	73	19.1
Gingivitis	68	17.8
Periodontitis	241	63.1
FMPI		
>1	259	67.8
≤1	123	32.2
Number of implants		
≥4	151	39.5
<4	231	60.5
Frequency of maintenance/year		
< 2	219	57.3
≥ 2	163	42.7

*FMPI* full-mouth plaque index

**Table 2:** Implant-related data analyzed in this study

Implant-related data	*N* = 1415	
	*n*	%
Dental arch		
Maxilla/mandibula	653/762	46.1/53.9
Position		
Maxilla anterior	169	11.9
Maxilla posterior	483	34.1
Mandibula anterior	219	15.5
Mandibula posterior	544	38.4
Keratinized tissue width		
< 2 mm	542	38.3
≥ 2 mm	873	61.7
Bone grafting		
Yes/No	214/1201	15.1/84.9
Periodontal diagnosis		
Healthy	204	14.4
Gingivitis	188	13.3
Periodontitis	1023	72.3
Type of prosthesis		
Removable/fixed	236/1179	16.7/83.3
Brand		
I	408	28.8
II	184	13.0
III	514	36.3
IV	188	13.3
V	121	8.6
Years from loading		
>24 months	1374	97.1

The mean age of the patients was 51.66 ± 12.16 years, ranging from 24 to 78 years. The mean follow-up was 3.79 ± 1.48 years (45.50 ± 17.78 months). Moreover, 97.1% (*n* = 1374) of implants had ≥ 2 years of follow-up, 70% (*n* = 990) had ≥ 3 years, and 41.1% (*n* = 582) had ≥ 4 years. The percentage of implants with more than 5 years of follow-up was 15% (*n* = 212).

In the medical history, systemic diseases were reported for 29.6% (*n* = 113) and diabetes were 11.8% (*n* = 45) of patients. Smokers were 34% (*n* = 130) of the sample. The majority of the patients (67.8%) had fair oral hygiene presenting a FMPI > 1 (*n* = 259), and 63.1% of the patients had periodontitis. Gingivitis was diagnosed in 17.8% of the study population, and 19.1% showed healthy tissues.

A total of 1415 implants were examined in this study: 46.1% in the maxilla and 53.9% in the mandible. The average PPD was 3.54 ± 1.53 mm (3.30 ± 0.91 for mucositis; 5.57 ± 1.44 for peri-implantitis), and the average PI was 1.44 ± 0.69 (1.66 ± 0.55 for mucositis; 1.84 ± 0.38 for peri-implantitis). The mean presence of bleeding was detected 50.49% (63.25% in mucositis and 73.19% in peri-implantitis). The keratinized tissue width around the implant was < 2 mm in 38.3% of the implants and also 3.9% of implants had absence of keratinized mucosa.

### Prevalence of peri-implant mucositis and peri-implantitis

Twenty-two percent of the patients (*n* = 84) showed healthy peri-implant mucosa. 41.1% (*n* = 157) of the patients presented peri-implant mucositis, whereas 36.9% (*n* = 141) exhibited peri-implantitis. Corresponding values were 12.6% (*n* = 19); 43.0% (*n* = 65); and 44.4% (*n* = 67) in subjects who had ≥ 4 number of implants.

Implant level analyses revealed that 24.7% of the implants (*n* = 350) presented no signs of peri-implant diseases. Furthermore, the prevalence of peri-implant mucositis and peri-implantitis at the implant level was 53.6% (*n* = 758) and 21.7% (*n* = 307), respectively (Table 3).

**Table 3:** Prevalence of peri-implant health and disease at patient and implant levels

	Patient level (*N* = 382)		Implant level (*N* = 1415)
	% (*n*)	Number of implants 1–3/≥4, % (*n*)	% (*n*)
Peri-implant health	22.0 (84)	28.1 (65)/12.6 (19)	24.7 (350)
Peri-implant mucositis	41.1 (157)	39.8 (92)/43.0 (65)	53.6 (758)
Peri-implantitis	36.9 (141)	32.0 (74)/44.4 (67)	21.7 (307)

### Risk indicators for peri-implant mucositis and peri-implantitis

#### Patient level

Table 4 depicts the ORs and 95% CI for each patient-based factor associated with peri-implant mucositis and peri-implantitis. According to the univariate regression analysis, age, frequency of maintenance, systemic disease, periodontal disease (periodontitis versus gingivitis/healthy), number of dental implants, and PI were significantly associated with peri-implant mucositis. In the multivariate regression model using these significant variables, frequency of maintenance (OR = 2.576, 95% CI 1.403–4.729; *p* = 0.002), periodontal disease (having periodontitis) (OR = 3.342, 95% CI 1.702–6.561; *p* = 0.000), and PI (OR = 3.046, 95% CI 1.724–5.383; *p* = 0.000) had significant associations with the development of peri-implant mucositis.

**Table 4:** Patient-related probable risk indicators for the occurrence of peri-implant mucositis and peri-implantitis

Variable		Peri-implant mucositis						Peri-implantitis					
		Univariate regression			Multivariate regression			Univariate regression			Multivariate regression		
		OR	95% CI	*p*	OR	95% CI	*p*	OR	95% CI	*p*	OR	95% CI	*p*
Gender	Female	0.673	(0.391–1.158)	0.153				0.577	(0.332–1.002)	0.051			
Age		1.056	(1.032–1.080)	0.000*				1.047	(1.023–1.071)	0.000*			
Smoking	Yes	1.179	(0.638–2.179)	0.599				2.981	(1.634–5.437)	0.000*			
Frequency of maintenance/year	< 2	2.866	(1.659–4.953)	0.000*	2.576	(1.403–4.729)	0.002*	2.278	(1.311–3.956)	0.003*	2.048	(1.074–3.907)	0.030*
Systemic disease	Yes	1.965	(1.021–3.782)	0.043				2.697	(1.353–5.021)	0.004*			
Diabetes	Yes	1.426	(0.491–4.147)	0.514				3.742	(1.382–10.136)	0.009*			
Periodontal disease	Periodontitis	5.874	(3.283–10.509)	0.000*	3.342	(1.702–6.561)	0.000*	6.634	(3.642–12.082)	0.000*	3.295	(1.593–6.815)	0.001*
Number of implants	>4	2.417	(1.324–4.412)	0.004*				3.097	(1.685–5.693)	0.000*	2.103	(1.040–4.251)	0.039*
PI		3.362	(2.038–5.546)	0.000*	3.046	(1.724–5.383)	0.000*	8.365	(4.769–14.670)	0.000*	7.055	(3.803–13.088)	0.000*

*PI* plaque index, *OR* odds ratio

*Indicates statistically significant

When the variables were analyzed with regard to peri-implantitis development at patient level, only gender did not reach statistical significance (OR = 0.577, 95% CI 0.332–1.002; *p* = 0.051). Instead, multivariate regression showed that PI was significantly associated with peri-implantitis development (OR = 7.055, 95% CI 3.803–13.088; *p* = 0.000). Furthermore, significant ORs were determined for patients having maintenance per year < 2 (OR = 2.048, 95% CI 1.074–3.907; *p* = 0.030), patients having ≥ 4 implants (OR = 2.103, 95% CI 1.040–4.251; *p* = 0.039), patients diagnosed with periodontitis (OR = 3.295, 95% CI 1.593–6.815; *p* = 0.001) via multivariate regression model.

#### Implant level

The implant-based variables associated with peri-implant mucositis and peri-implantitis are summarized in Table 5. The significant variables obtained by univariate regression were utilized for multivariate regression. Accordingly, having a systemic disease (ORs = 2.196/3.789), diagnosed as periodontitis (ORs = 6.931/18.613), keratinized tissue width < 2 mm (ORs = 5.389/8.013), PPD (ORs = 1.570/8.338), PI (ORs = 6.726/5.205), and BoP (ORs = 3.645/4.353) independent variables were significantly associated with both peri-implant mucositis and peri-implantitis, respectively.

**Table 5:** Dental implant-related probable risk indicators for the occurrence of peri-implant mucositis and peri-implantitis

Variable		Peri-implant mucositis						Peri-implantitis					
		Univariate regression			Multivariate regression			Univariate regression			Multivariate regression		
		OR	95% CI	*p*	OR	95% CI	*p*	OR	95% CI	p	OR	95% CI	*p*
Gender	Female	0.606	(0.468–0.785)	0.000*				0.527	(0.386–0.720)	0.000*			
Smoking	Yes	1.960	(1.458–2.634)	0.000*				3.413	(2.430–4.793)	0.000*			
Frequency of maintenance	< 2/year	2.076	(1.605–2.684)	0.000*				2.020	(1.478–2.760)	0.000*			
Systemic disease	Yes	2.301	(1.663–3.185)	0.000*	2.196	(1.090–4.427)	0.028*	3.058	(2.116–4.419)	0.000*	3.789	(1.667–8.614)	0.001*
Diabetes	Yes	3.741	(1.770–7.907)	0.001*				4.460	(2.007–9.911)	0.000*			
Periodontal Diagnosis	Periodontitis	3.666	(2.792–4.814)	0.000*	6.931	(3.303–14.544)	0.000*	4.336	(3.038–6.190)	0.000*	18.613	(7.304–47.427)	0.000*
Jaw	Upper	0.586	(0.454–0.758)	0.000*				0.433	(0.316–0.592)	0.000*			
Position	Upper anterior	1.205	(0.816–1.779)	0.348*				0.747	(0.447–1.250)	0.267			
	Upper posterior	0.512	(0.394–0.664)	0.000*				0.474	(0.343–0.655)	0.000*			
	Lower anterior	2.945	(1.885–4.601)	0.000*				2.775	(1.680–4.583)	0.000*			
	Lower posterior	1.106	(0.849–1.440)	0.454				1.487	(1.086–2.037)	0.013*			
KT < 2 mm	Yes	8.819	(5.841–13.316)	0.000*	5.389	(2.637–11.010)	0.000*	17.439	(11.133–27.315)	0.000*	8.013	(3.494–18.380)	0.000*
PPD		4.806	(3.882–5.951)	0.000*	1.570	(1.105–2.230)	0.012*	24.837	(18.477–33.385)	0.000*	8.338	(5.540–12.548)	0.000*
PI		30.209	(20.120–45.357)	0.000*	6.726	(3.829–11.812)	0.000*	51.175	(32.298–81.083)	0.000*	5.205	(2.570–10.541)	0.000*
GI	≥ 2	13.870	(10.685–18.004)	0.000*				40.955	(15.184–110.467)	0.000*			
BoP		4.665	(3.860–5.637)	0.000*	3.645	(2.817–4.716)	0.000*	6.046	(4.911–7.444)	0.000*	4.353	(3.239–5.851)	0.000*
Bone augmentation	Yes vs. No	0.979	(0.691–1.388)	0.905				0.850	(0.550–1.313)	0.464			
Type of prosthesis	Removable vs. fixed	3.744	(2.369–5.917)	0.000*				3.103	(1.857–5.186)	0.000*			
Years from loading		0.992	(0.985–0.999)	0.023*				0.995	(0.987–1.003)	0.240			
Brand	I	0.921	(0.694–1.221)	0.565				1.281	(0.920–1.784)	0.143			
	II	1.064	(0.749–1.512)	0.727				0.189	(0.094–0.378)	0.000*			
	III	1.019	(0.784–1.323)	0.890				0.761	(0.550–1.052)	0.098			
	IV	1.077	(0.709–1.636)	0.729				2.757	(1.780–4.272)	0.000*			
	V	0.965	(0.617–1.511)	0.877				0.912	(0.526–1.582)	0.744			

*KT* keratinized tissue, *PPD* probing pocket depth, *PI* plaque index, *GI* gingival index, *BoP* bleeding on probing, *OR* odds ratio

*Indicates statistically significant

## Discussion

The present non-interventional study evaluated the frequency of peri-implant diseases and probable risk indicators of both peri-implant mucositis and peri-implantitis in patients applied to a university periodontology department.

This study included a total of 382 subjects, with 1415 implants. Peri-implant mucositis was defined as the presence of BoP without bone loss after initial remodeling as suggested and as in line with the literature [1, 20, 21]. But the definition of peri-implantitis vary across the studies. Some studies diagnose peri-implantitis according to the bone level changes with reference to the exposed implant thread [22–24]; others agree for the marginal bone loss in millimeters ranging from 0.5 to 5 mm as threshold [2, 3, 25, 26]. Therefore, different case definitions and different thresholds cause wide range of the prevalences of peri-implant diseases. In our study, the peri-implantitis was diagnosed as having > 2 mm marginal bone loss, > 5 mm PPD with BoP and the prevalence of patients presenting peri-implant mucositis and peri-implantitis were 41.1% and 36.9%, respectively. The prevalence increases to 43.0% for peri-implant mucositis and 44.4% for peri-implantitis in patients having number of implants ≥ 4. When we analyze the results at implant levels, the prevalences were determined as 53.6% and 21.7% for peri-implant mucositis and peri-implantitis, respectively. These results are similar to some studies [26–29]. On the other hand, compared to other studies, these prevalences are relatively high in which the prevalence of peri-implantitis ranges from 1–15.8% [2, 25, 30–32] at patient level. These different results could be attributed to the characteristics of the study population including having different probable risk factors for peri-implant diseases development and also the different case definitions. Furthermore, reporting the prevalences as subject or implant level is making it difficult to compare the results with the literature. But, general opinion is that the prevalences of peri-implant diseases should be assessed at subject level as in other chronic systemic diseases [20].

Based on the results of multivariable multinomial regression analysis, frequency of maintenance < 2/year (OD = 2.57; 2.04), patients diagnosed as periodontitis (OD = 3.31; 3.29), and PI (OD = 3.04; 7.05) were associated with peri-implant mucositis and peri-implantitis development at patient level, respectively. In addition to these results, patients who have ≥ 4 number of implants have higher risk for peri-implantitis occurrence (OD = 2.10) than patients having number of implants < 4 as reported in numerous studies [3, 22, 33–35]. These results are in agreement with the literature as follows: higher PI (which also demonstrates poor oral hygiene) [27, 30, 36], patients who do not participate to the maintenance therapy [7, 37], and patients who had history of periodontitis were demonstrated as risk indicators for the development of peri-implant disease [4, 33, 38]. In this cross sectional study, patients who have current diagnosis of periodontitis had increased probability for development of peri-implant disease. Therefore, our results are significant to reveal the importance of current periodontal status as a risk indicator.

When the putative risk indicators for peri-implant disease were analyzed at implant level, it was determined that, in addition to PI and periodontitis diagnosis, higher PPD, and BoP, having a systemic disease and keratinized tissue (KT) width < 2 mm were associated with both peri-implant mucositis and peri-implantitis occurrence. Among these variables, PI, PPD, BoP, and current periodontitis diagnosis are relatively predicted variables, but systemic disease and KT width are controversial. In our study, patients with systemic diseases (cardiovascular disease, hypertension, and diabetes) have increased risk for mucositis (OD = 2.1) and peri-implantitis (OD = 3.7) development than patients who were systemically healthy. On the other hand, when diabetes was analyzed solely as an indicator rather than having a systemic disease, multivariate anayses did not find an association with the peri-implant disease development. Conversely, Monje et al. [39] reported that patients with diabetes were at high risk for peri-implantitis development but not for peri-implant mucositis. Furthermore, implant survival rates were reported as high in patients who were taking antihypertensive drugs than systemically healthy subjects [40]. In our study, drug usage could not be analyzed because of presenting multicolinerity with the variable sytemic disease data. These results could be attributed to the patients with diabetes and might be relatively more under controlled than patients with HT and having cardiovascular disease. Besides, self-reported patients’ medical history was recorded. The hemoglobin A1c (HbA1c) levels were not measured in patients with diabetes.

In the literature, there is no consensus about the KT width around dental implant, whether necessary or not. Additionally, the adequate width of KT is controversial. Several researchers reported that KT is not necessary to maintain peri-implant tissue health [11, 12]. Conversely, in line with our results, the necessity of KT around implant-supported prostheses have been shown to be essential for proper plaque control [41]. Unexpectedly, Pimentel et al. [35] and Roos-Jansaker et al. [42] demonstrated that presence of KT is associated with peri-implant inflammation. In our study, implants with KT < 2 mm have greater change for peri-implant mucositis (OD = 5.38) and peri-implantitis (OD = 8.01) development respectively, than implants having KT ≥ 2 mm. In our study, although the amount of bone loss was not evaluated, it is assumed that less KT is more related to the plaque accumulation rather than the bone loss around implants because higher PIs were scored in implants having KT < 2 mm as reported in other studies [41]. The different results about the importance of KT among studies might be due to the differences in the thereshold values of KT and follow-up times considered. Furthermore, the characteristics of the study population and also the different probable risk indicators included in the analysis could be the reasons.

PPD was determined as another risk indicator for peri-implant disease development in this study. This result was first reported by Vignoletti et al. [3]. They revealed that implants having PPD ≥ 4 mm had increased risk for peri-implantitis development (OD = 3.5). In our cross-sectional study, for each 1 mm increase in PPD, the OR of having peri-implant mucositis increase was 1.57, and peri-implantitis increase was 8.33. In the literature, increased PPD was associated with increased BoP [43] and bone loss [44]. Furthermore, increased PPD would ensure anaerobic microbial growth and this would cause soft and hard tissue breakdown [45]. On the other hand, PPD is an important criteria for diagnosis of peri-implant diseases. Therefore, PPD, which was identified as a risk indicator, should be verified in the prospective studies because PPD might be a consequence rather than a risk due to the cross-sectional nature of our study.

The results of this cross-sectional study might have been effected by some limitations. Firstly, the occlusal overload and some possible risk indicators as platform switching related to the implant supported prosthesis were not evaluated. Second, patients who had given up smoking were accepted as non-smokers and only the current smoking status of the patients were considered. Therefore, this might have masked the actual effect of smoking on the development of peri-implant diseases. Third, the cross-sectional nature of our study only allows us to consider one evaluation time period rather than giving information about the progression of the disease. Thus, this limits us to make interpretation about the causality. Besides these limitations, the multilevel multivariable analyses and also both the patient- and implant-level analyses including numerous variables are the strengths of our study.

## Conclusıons

Within the limitations of the present study, a high prevalence of peri-implant diseases were determined in a Turkish population rehabilitated in a university clinic. Higher probabilities of peri-implantitis were detected in patients having maintenance < 2/year, presence of periodontitis, poor plaque control, and having number of implants ≥4. Systemic disease, less keratinized tissue (< 2 mm), PPD, and BoP were also risk indicators for peri-implantitis development.

## Data Availability

The datasets used and analyzed during the current study are available from the corresponding author on reasonable request.

## Abbreviations

BoP: Bleeding on probing
KM: Keratinized mucosa
PPD: Probing pocket depth
PI: Plaque index
GI: Gingival index
OR: Odds ratio
CI: Confidence intervals
KT: Keratinized tissue
